# Association between Thigh Muscle Volume and Leg Muscle Power in Older Women

**DOI:** 10.1371/journal.pone.0157885

**Published:** 2016-06-17

**Authors:** Ulrich Lindemann, Christian Mohr, Juergen Machann, Konstantinos Blatzonis, Kilian Rapp, Clemens Becker

**Affiliations:** 1 Department of Clinical Gerontology and Rehabilitation, Robert-Bosch-Hospital, Stuttgart, Germany; 2 Institute for Diabetes Research and Metabolic Diseases (IDM) of the Helmholtz Center Munich at the University of Tübingen, Tübingen, Germany; 3 Department of Diagnostic and Interventional Radiology, Section on Experimental Radiology, University Hospital Tübingen, Tübingen, Germany; 4 Department of Radiology and Nuclear Medicine, Robert-Bosch-Hospital, Stuttgart, Germany; Universidad Pablo de Olavide, Centro Andaluz de Biología del Desarrollo-CSIC, SPAIN

## Abstract

The construct of sarcopenia is still discussed with regard to best appropriate measures of muscle volume and muscle function. The aim of this post-hoc analysis of a cross-sectional experimental study was to investigate and describe the hierarchy of the association between thigh muscle volume and measurements of functional performance in older women. Thigh muscle volume of 68 independently living older women (mean age 77.6 years) was measured via magnetic resonance imaging. Isometric strength was assessed for leg extension in a movement laboratory in sitting position with the knee flexed at 90° and for hand grip. Maximum and habitual gait speed was measured on an electronic walk way. Leg muscle power was measured during single leg push and during sit-to-stand performance. Thigh muscle volume was associated with sit-to-stand performance power (r = 0.628), leg push power (r = 0.550), isometric quadriceps strength (r = 0.442), hand grip strength (r = 0.367), fast gait speed (r = 0.291), habitual gait speed (r = 0.256), body mass index (r = 0.411) and age (r = -0.392). Muscle power showed the highest association with thigh muscle volume in healthy older women. Sit-to-stand performance power showed an even higher association with thigh muscle volume compared to single leg push power.

## Introduction

The loss of muscle function and mass in the ageing population is increasingly recognized as relevant, common and modifiable. Depending on the definition, the prevalence of sarcopenia in older persons 60–80 years old is often reported in the range of 5–13% [1]. Sarcopenia is increasing to more than 30% in persons 80 years old and older [2]. Many common medical conditions such as stroke, hip fracture and diabetes mellitus and medications like corticosteroids or α-blockers increase the incidence of sarcopenia [3–5]. Public bodies and authorities such as the World Health Organisation, the United States National Institute of Health, the European Medicines Agency and the United States Food and Drug Administration are currently discussing different definitions for sarcopenia. One major point is the question whether sarcopenia should be recognized as a disease entitiy in the revised International Classification of Diseases and Related Health Problems or as a syndrome. An even bigger challenge is the definition of sarcopenia. A European formalized consensus group has made an attempt to come up with a clinical definition for screening [6]. A key issue of a definition is the relationship between muscle mass, impairment and function. There are considerable disparities in studies how and where to measure muscle mass, muscular impairment and functional loss caused by sarcopenia. Many studies have pragmatically measured total body muscle mass using dual-energy X-ray absorptiometry (DXA) with limited accuracy. At the same time muscle function was measured looking at lower extremity function such as habitual gait speed or sometimes hand grip strength as a surrogate marker. The pharmaceutical industry has started trials for the development and evaluation of new medications [7] and non-pharmaceutical interventions [8,9]. In summary, effective treatments to prevent, slow and/or reverse the progression of sarcopenia could be very relevant to influence ageing trajectories. However, in order to assess incidence, prevalence and treatment effects accurate measures are required to measure, screen, assess and follow older persons and certain patient groups. This requires methods to quantify muscle mass and relevant functional measures.

The current gold standard to measure muscle mass is magnetic resonance imaging (MRI) [10]. For muscle mass screening and other purposes other methods such as DXA or bioelectrical impedance analysis (BIA) may be required but their limitations must be realized. To measure muscle strength, power and functional loss caused by sarcopenia several methods have been established and are widely accepted but often not vigorously applied. There is consensus that muscle strength parameters, such as hand grip strength and isometric measurements of the lower extremity, should be measured. Functional performance tests, such as the assessment of chair rise time and gait speed are required and accepted to study the effects of sarcopenia on the loss of independence. All of these tests have been instrumented to increase their accuracy and objectivity [11,12]. Although leg muscle power has been identified as an important determinant of mobility skills in older adults [13] and although leg muscle power has been shown to be associated with muscle volume in young persons [14], at present this measure is often not used to show the efficacy of strategies to counteract sarcopenia. The standard equipment to measure muscle power is the Nottingham Power Rig, where power is calculated from a single leg push in a seated position (Bassey & Short, 1990). Recently it has been shown that the sit-to-stand (STS) power performance, as a functional measure of the lower extremity, can objectively be assessed in older persons by a linear encoder [15,16] or by body-worn sensors [17]. The measurement of muscle power is often neglected even though the relevance is obvious [13,18,19]. The association between muscle mass, strength, function and power is still understudied. Often, authors implicitly state that these measures can be used as surrogate markers interchangingly. While this might be justified for epidemiological studies or sometimes in clinical practise it is certainly questionable in efficacy studies on new medications and non-pharmaceutical RCTs.

The aim of this post-hoc analysis was to investigate and describe the hierarchy of the association between thigh muscle volume and measurements of functional performance in older women. In particular we were interested in the consensus based use of hand grip strength and habitual gait speed to screen for sarcopenia and to compare these with measures of muscle power. We hypothesized that leg muscle power is a potent measure in this hierarchy.

## Methods

### Subjects and design

Sixty-eight older women (mean age 77.6 years) were included into this cross-sectional post-hoc analysis. The original study (DRKS00003622) was to compare physical performance in a 15°C and a 25°C temperature indoor environment [20]. Data from the 25°C temperature condition were used for this post-hoc analysis. From the original data set of 93 subjects 25 data sets were excluded, because the pre-defined margins of the thigh muscle volume, which were used for muscle volume analysis via MRI, were not precisely detectable, e.g. because of prostheses. Inclusion criteria of the original study were: age ≥ 70years, female gender and community dwelling. Women with uncontrolled cardiac illness or a relevant functional impairment due to neurological and/or orthopedic diseases that significantly influenced transfers or walking performance were excluded from the study. Cognitive impairment based on screening via the Short Orientation Memory Concentration Test [21] with a cut-off score >10 (worst score 28) was another exclusion criterion. Comorbidity was described using the Functional Comorbidity Index [22]. The body mass index (BMI) was calculated from body mass and body height and was used as a descriptive parameter and for analysis.

The study was conducted according to the principles expressed in the Declaration of Helsinki. Prior to first assessment all participants gave written informed consent to participate in the study. The study was approved by the ethics committee of the medical faculty of the university of Tuebingen, Germany (578/2011BO2). The individual in this manuscript, shown in a figure, has given written informed consent (as outlined in PLOS consent form) to publish these case details.

### Muscle volume imaging

**Fig 1:**
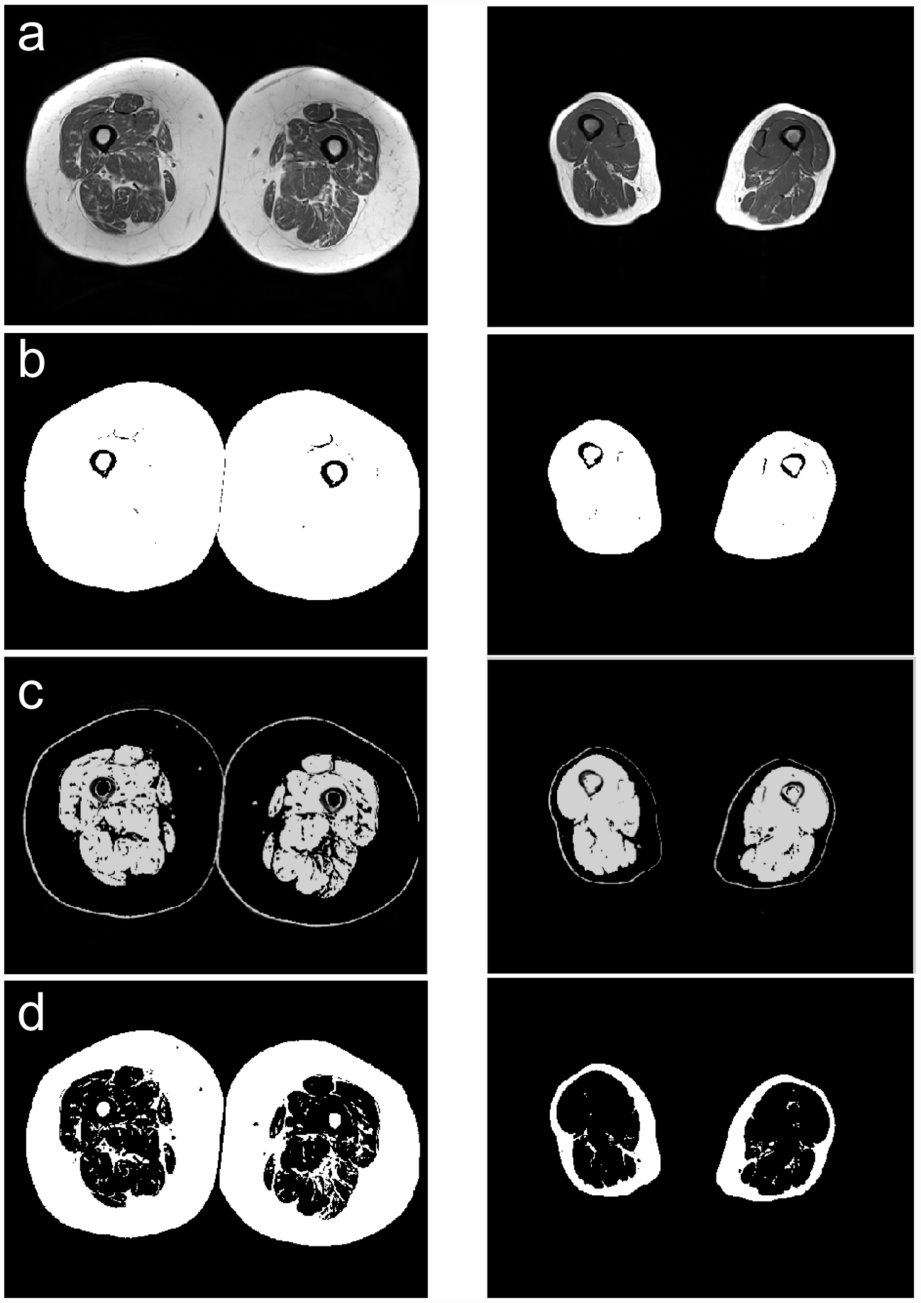
Representative axial slice from the upper thigh with (a) original image, (b) total tissue in the cross section, (c) segmented lean tissue, i.e. skeletal muscle, (d) segmented adipose tissue.

Muscle volumes of both thighs were assessed using MRI (MAGNETOM Aera1.5 T, Siemens Healthcare, Erlangen, Germany). Subjects were lying in supine position on the spine-array coil and were covered by an 8-channel body array-coil of the manufacturer. Imaging was performed from trochanter minor down to the transition of the muscle rectus femoris to its tendon applying an axial T1-weighted fast spin-echo technique. Measurement parameters: TE = 9.8ms, TR = 567ms, matrix size 346x512, in-plane resolution 0.8x0.9 mm, acquisition time 3:08 min. with a slice thickness of 8 mm and without interslice gap. A home-built semi-automatic segmentation program based on Matlab (Matlab 8.4, the MathWorks Inc., Natick, Massachusetts, USA) was used for determination of muscle volume. This threshold-based algorithm segments three classes: noise, lean tissue (i.e. muscle) and adipose tissue as exemplary shown in Fig 1. Pixels in the respective class were summed and the cross-section was calculated by multiplying the number of pixels by the in-plane resolution. Summing up all slices resulted in total volume of lean tissue and adipose tissue.

In a second step the left and the right thigh muscles were separated by manually drawing a region of interest covering the left and right thigh muscle cross-section, respectively, in each slide. The total muscle volume resulted in summing up all slides and multiplying by scan thickness. The values [cm^3^] of both thighs and the sum were used for analysis.

### Muscle parameters of functional performance

#### Muscle strength

Maximum quadriceps strength was assessed using a hand-held dynamometer (microFET2, Biometrics, Almere, The Netherlands) in a sitting position with the knee flexed at 90°. Participants were instructed to stretch their knee as much as possible. After measuring the lever distance (knee joint—application), the sum of the maximum force momentum [Nm] from a single trial of each leg was used for analysis.

Maximum hand grip strength [kg] was assessed with a dynamometer (Jamar, MSD, Londerzeel, Belgium, respectively). The value of a single trial with the preferred hand was used for analysis.

#### Muscle function

Maximum leg muscle power was assessed using the Nottingham Power Rig [23]. Subjects were seated with the pelvis supported at the back. They were instructed to push as hard as possible against a plate with a single leg over a distance of 16.5 cm. Power was inferred from the angular velocity and inertia of a flywheel, which was driven by a chain connected to the foot plate by a lever. After two sub-maximal trials of each leg, the maximum results for left and right leg power [W], out of three trials for each leg, were used seperately for analysis. Additionally, the maximum results were added and used for analysis.

**Fig 2:**
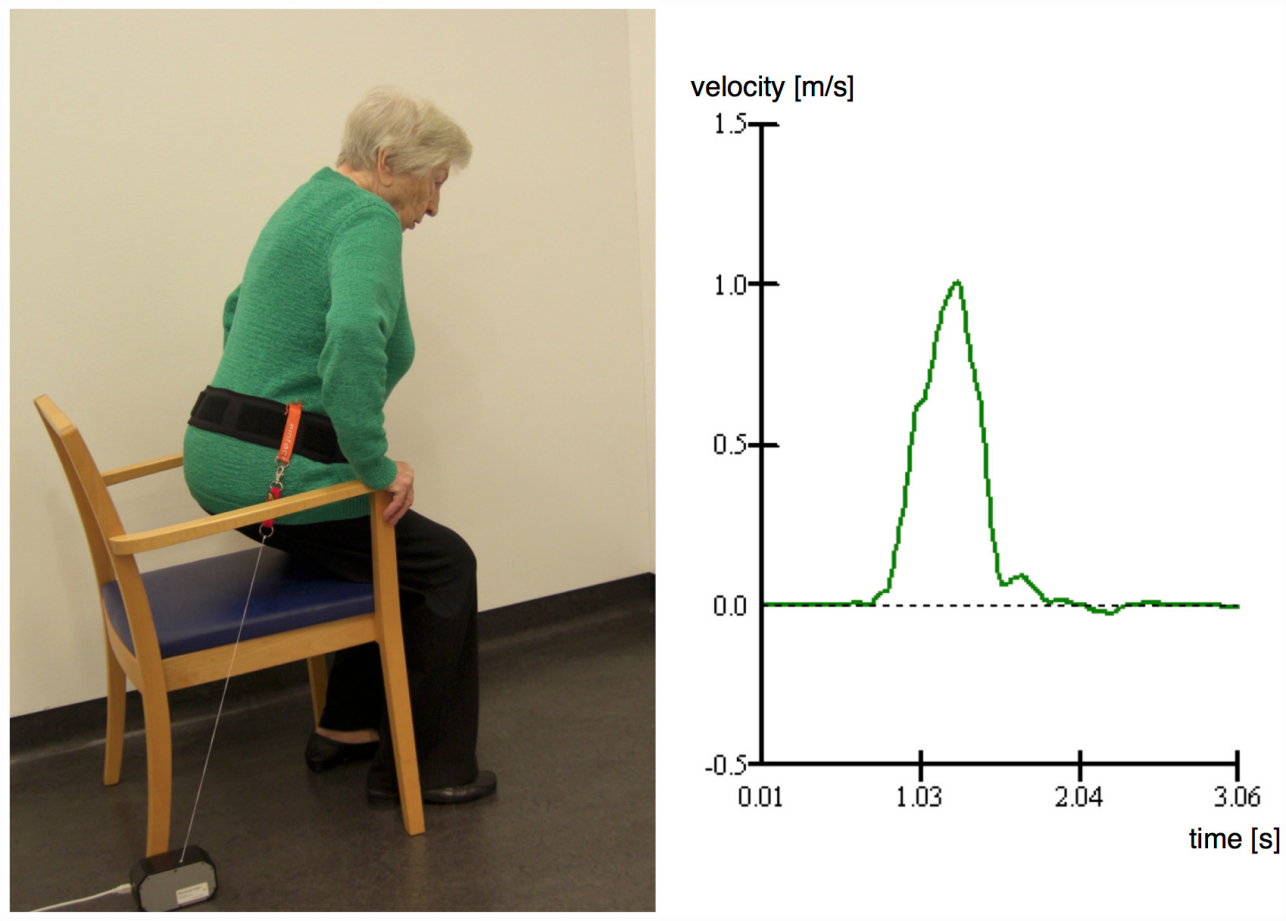
Linear encoder fixed at the person’s hip measuring the velocity of the hip as a function of time.

Participants were asked to stand up from a standard chair (46 cm) with armrests as quickly as possible to the full standing position in order to measure the STS performance. A linear encoder (MuscleLab Powermodel MLPRO, Ergotest Technology, Langesund, Norway) was fixed at the person’s hip near vertebra L5 with a waist belt and measured the velocity of the hip as a function of time (Fig 2). After demonstration of the task and one sub-maximal trial STS performance power [W] was calculated from the hips´ maximum velocity multiplied by body weight [16] and was used for analysis.

Gait speed was assessed over a distance of 8 m with an additional 3 m for acceleration and deceleration [24]. Spatiotemporal gait parameters were recorded on an instrumented walkway with embedded pressure sensors (GAITRite, CIR Systems, Haverton, PA, USA), and calculated by application software. The use of a walking aid was allowed. The instructions were to walk along the walk-way once at ‘normal speed’ and once ‘as fast as possible, but safely’. Habitual and maximum gait speeds [m/s] were used for analysis.

### Statistical analysis

The associations between variables were expressed using Pearson’s coefficient of correlation. In addition, a linear regression model was conducted to analyze the independent and combined contribution of the possible determinants of thigh muscle volume. Parameters were included, if the association with muscle volume was r>0.4. All analyses were conducted using SPSS Version 16 (IBM Corp., Armonk, USA).

## Results

The mean age of the women was 77.6 years (SD 5 years) and their mean habitual gait speed was 1.1 m/s (SD 0.22 m/s) with 6 women using a walking aid. The cohort is described in more detail in Table 1.

**Table 1:** Descriptive data of all (n = 68) included women.

	Mean (SD)	Range
**Age [years]**	77.6 (4.99)	70–89
**Height [cm]**	160 (6.26)	145–173
**Body mass [kg]**	68.5 (11.88)	44–102
**BMI [kg/m**^**2**^**]**	26.9 (4.17)	17–38
**Comorbidity (****0****–18)**	2.7 (1.68)	0–6
**Cognition (****0****–28)**	2.6 (3.06)	0–10
**Total thigh muscle volume [cm**^**3**^**]**	4021 (684.98)	2768–5639
**Left thigh muscle volume [cm**^**3**^**]**	2068 (351.62)	1421–2960
**Right thigh muscle volume [cm**^**3**^**]**	1953 (347.83)	1346–2906
**Sit-to-stand power [W]**	727 (174.63)	365–1161
**Nottingham Power Rig, both legs [W]**	213 (73.08)	123–464
**Nottingham Power Rig, left leg [W]**	111 (38.61)	47–232
**Nottingham Power Rig, right leg [W]**	102 (36.70)	48–235
**Habitual gait speed [m/s]**	1.10 (0.22)	0.49–1.54
**Maximum gait speed [m/s]**	1.76 (0.34)	0.73–2.39
**Quadriceps strength [Nm]**	126 (33.39)	56–232
**Hand grip strength [kg]**	24.4 (4.12)	15–33

BMI = Body Mass Index; Comorbidity assessed by the Functional Comorbidity Index; Cognition assessed by the Short Orientation Memory Concentration Test; better score values are underlined

**Fig 3:**
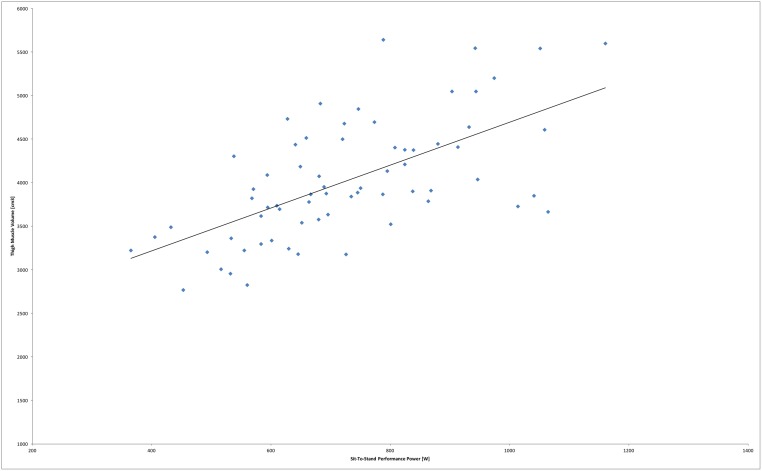
Association between thigh muscle volume of both legs with Sit-To-Stand performance power for all (n = 68) participating women, including a trend line (r = 0.628).

The highest associations between thigh muscle volume and other parameters were found for STS performance power (r = 0.628; Fig 3) and for leg push power sum value (r = 0.550).

Somewhat lower correlations were observed for isometric quadriceps strength (r = 0.442). The association between thigh muscle volume and hand grip strength (r = 0.367), fast gait speed (r = 0.291) and habitual gait speed (r = 0.256) were even lower in this sample.

Total thigh muscle volume was strongly associated with single left and right muscle volume (r = 0.980 and r = 0.979, respectively). Coefficients of correlation describing the single leg association between thigh muscle volume and leg push power were r = 0.574 for left leg and r = 0.431 for right leg. The associations between thigh muscle volume and body mass index and age were r = 0.411 and r = -0.392, respectively. A linear regression model including STS performance power, isometric strength and body mass index as independent variables explained 50% of the variance of thigh muscle volume.

The coefficients of inter-correlation between parameters of functional performance ranged from r = -0.142 to r = 0.608 and are shown in detail in Table 2.

**Table 2:** Association between total thigh muscle volume and parameters of functional performance described by Pearson´s coefficient of correlation.

	STS power	Power Rig (sum value)	Gait speed (maximum)	Gait speed (habitual)	Quadriceps strength	Hand grip strength
**Thigh muscle volume (total)**	0.628	0.550	0.291	0.256	0.442	0.367
**Sit-To-Stand power**		0.608	0.507	0.173	0.404	0.409
**Power Rig (sum value)**			0.500	0.147	0.607	0.490
**Gait speed (maximum)**				0.319	0.370	0.344
**Gait speed (habitual)**					0.172	-0.142
**Quadriceps strength**						0.476

## Discussion

In this group of relatively healthy older women we found a moderate association between thigh muscle volume and muscle power. These results are corroborated by a study with younger subjects [14]. The highest association between muscle thigh volume and functional performance was found for power measured during the STS transfer.

The low association between thigh muscle volume and hand grip strength as well as habitual gait speed questions the usefulness of these parameters to screen older persons for sarcopenia [19,6]. It seems problematic to use these parameters as surrogate markers for outcome studies, such as RCTs with pharmaceutical interventions. This is not questioning the use of gait speed and other parameters as outcome parameters for other purposes.

Most studies currently include standardized measurements for hand grip strength and gait speed. Power measurements are performed less frequently although the relevance has been recognized since many years [13]. The reason for excluding power is mostly related to the cost and space requirements of the equipment. The recommendation to use hand grip strength as a functional outcome measure of sarcopenia was based on similar results for leg power, assessed by the Nottingham Power Rig, and hand grip strength to predict poor walking performance, but better practicability of hand grip strength [19]. In a recent study, stair climbing, chair rise and fast gait speed were affected by a myostatin inhibitor intervention to increase muscle mass [25]. Since all these parameters assess some kind of strength and distance per time, which describes “power”, the high association between thigh muscle volume and STS performance power in our study is reasonable. Simple and objective measures of muscle power are available now [15,16] and therefore we included this method. This was found to have the strongest correlation with thigh muscle volume. When measuring muscle power during the STS transfer body weight and height are major factors which is different from the power rig assessment which is performed in a sitting position. Inclusion of body mass index into the regression model to predict muscle volume takes into account the obvious influences of body mass and height. Furthermore and with regard to functional use of legs, punctum fixum and punctum mobile are transposed during assessment of leg push power via Nottingham Power Rig. Our results of higher association between functional assessment of muscle power and thigh muscle volume compared to lower association between sitting leg push power and thigh muscle volume are corroborated by studies of Wilhelm et al. [26]. They found higher association between counter-movement jump power and thigh muscle quality (echo intensity) compared to lower association between leg extensor power and thigh muscle quality.

In our study, the muscle volume was calculated via MRI. In contrast to the use of DXA, we have chosen MRI, which has no radiation exposure. DXA measures the full body muscle volume, whereas our method only assessed the leg muscle volume. This makes it possible to better match the leg power with the muscle volume of the thigh. On the other hand, the hand grip strenght is not mapped directly in the muscle volume values. The measurement of muscle volume via MRI and the MATLAB based programm allows a detailed analysis of the real muscle volume but is more complex, costly and time consuming than DXA. With regard to detection of sarcopenia it must be questioned which muscle groups are most relevant [27] and whether these muscle groups can be better measured via DXA or via MRI. In previous studies it has been shown that there is an expected correlation between the results of DXA and MRI assessed muscle volume, but that DXA underestimates the muscle volume [28]. With regard to controlled studies to improve muscle volume, small but relevant improvement of muscle volume may not be detected or underestimated by DXA-measurement due to limited accuracy.

The history of the discussion on osteoporosis indicates that key zones for definitions are preferable for the diagnosis and for consented definitions [29,30]. In the case of sarcopenia this means that if lower extremity function, muscle strength and power are used for diagnosis, imaging should be done with an accurate measure in the same region. This is currently not addressed in the ongoing consensus discussions.

A limitation of our study is that the results are valid only for this cohort of healthy older women. Future and preferably prospective studies should evaluate our findings in a larger sample size of frail older persons and should explicitly include men.

In conclusion, power showed the highest association with thigh muscle volume in healthy older women. The power measurement of STS performance showed an even higher association with thigh muscle volume compared to the measurement via Nottingham Power Rig. This should be discussed in the debate on screening tools and outcome measurements for sarcopenia.

## Supporting Information

S1 Raw Data contains the complete data set. (XLSX)
